# Role of Routine Suppressive Antibiotic Therapy After Debridement, Antibiotics, and Implant Retention for Acute Periprosthetic Joint Infections

**DOI:** 10.1093/ofid/ofae216

**Published:** 2024-04-17

**Authors:** Don Bambino Geno Tai, Aaron J Tande, Benjamin Langworthy, Matthew P Abdel, Elie F Berbari, Bas ten Have, Paul Jutte, Alex Soriano, Gina A Suh, Wierd Zijlstra, Marjan Wouthuyzen-Bakker

**Affiliations:** Division of Infectious Diseases and International Medicine, Medical School, University of Minnesota, Rochester, Minnestota, USA; Division of Public Health, Infectious Diseases and Occupational Medicine, Mayo Clinic, Rochester, Minnestota, USA; Division of Public Health, Infectious Diseases and Occupational Medicine, Mayo Clinic, Rochester, Minnestota, USA; Division of Biostatistics and Health Data Science, School of Public Health, University of Minnesota, Rochester, Minnestota, USA; Department of Orthopedic Surgery, Mayo Clinic, Rochester, Minnestota, USA; Division of Public Health, Infectious Diseases and Occupational Medicine, Mayo Clinic, Rochester, Minnestota, USA; Department of Orthopedic Surgery, Martini Hospital, Groningen, the Netherlands; Department of Orthopedic Surgery, University Medical Center Groningen, University of Groningen, Groningen, the Netherlands; Department of Infectious Diseases, Hospital Clinic of Barcelona, University of Barcelona, CIBER in Infectious Diseases (CIBERINFEC), Spain; Division of Public Health, Infectious Diseases and Occupational Medicine, Mayo Clinic, Rochester, Minnestota, USA; Department of Orthopedic Surgery, Medical Center Leeuwarden, Leeuwarden, the Netherlands; Department of Medical Microbiology and Infection Prevention, University Medical Center Groningen, University of Groningen, Groningen, the Netherlands

**Keywords:** chronic suppression, DAIR, implant retention, prosthetic joint infections, suppressive antibiotic therapy

## Abstract

**Background:**

The first-line management strategy for acute periprosthetic joint infections (PJIs) is debridement, antibiotics, and implant retention (DAIR). Suppressive antibiotic therapy (SAT) after DAIR is proposed to improve outcomes, yet its efficacy remains under scrutiny.

**Methods:**

We conducted a multicenter retrospective study in patients with acute PJI of the hip or knee who were treated with DAIR in centers from Europe and the United States. We analyzed the effect of SAT using a Cox model landmarked at 12 weeks. The primary covariate of interest was SAT, which was analyzed as a time-varying covariate. Patients who experienced treatment failure or were lost to follow-up within 12 weeks were excluded from the analysis.

**Results:**

The study included 510 patients with 66 treatment failures with a median follow-up of 801 days. We did not find a statistically significant association between SAT and treatment failure (hazard ratio, 1.37; 95% CI, .79–2.39; *P* = .27). Subgroup analyses for joint, country cohort, and type of infection (early or late acute) did not show benefit for SAT. Secondary analysis of country cohorts showed a trend toward benefit for the US cohort (hazard ratio, 0.36; 95% CI, .11–1.15; *P* = .09), which also had the highest risk of treatment failure.

**Conclusions:**

The utility of routine SAT as a strategy for enhancing DAIR's success in acute PJI remains uncertain. Our results suggest that SAT's benefits might be restricted to specific groups of patients, underscoring the need for randomized controlled trials. Identifying patients most likely to benefit from SAT should be a priority in future studies.

## Teaser

An international study on suppressive antibiotic therapy after debridement, antibiotics, irrigation, and implant retention for periprosthetic joint infection revealed uncertain benefits, questioning its routine use and emphasizing the need for further research.

Periprosthetic joint infections (PJIs) are challenging infections to manage, even among experienced orthopedic surgeons and infectious disease doctors. A widely accepted management strategy for acute PJI is debridement, antibiotics, and implant retention (DAIR). DAIR is less invasive than complete resection of the prosthesis, although the outcomes are poorer. Treatment success rates hover around 60% to 67%; this is typically defined as clinical infection eradication, no need for further surgery, no PJI-related mortality, and no use of suppressive antibiotic therapy (SAT) [1]. The poor outcomes are partly due to the presence of biofilm, rendering them refractory to the host immune system and antibiotic therapy [2]. Therefore, improving DAIR's success rates is paramount among clinicians and patients.

A persistent question in DAIR is the role and efficacy of SAT. SAT is generally a long-term antimicrobial treatment intended to prevent the recurrence of infection. Practices differ around the world, as previous studies have shown mixed results regarding its effectiveness in preventing PJI recurrence post-DAIR [3]. The ambiguity surrounding the benefits and potential drawbacks of SAT stems from a lack of comprehensive high-quality randomized controlled trials. Furthermore, the existing literature predominantly consists of studies with a small sample size and single-center studies with varied definitions of infection and outcomes [4, 5]. The best evidence in the duration of antibiotic therapy in DAIR is guided by the DATIPO trial, which showed that 6 weeks of therapy is not noninferior to 12 weeks [6]. In this analysis, patients treated with 12 weeks of antibiotic therapy after DAIR still had a failure rate of 14.5%, which raises the question whether patients might benefit from an even longer duration of treatment.

The primary aim of our research is to investigate the utility of SAT following acute PJI managed with DAIR in a cohort of patients in which the prescription of SAT is routine practice (United States) and in a cohort in which SAT is prescribed only in a select population (Europe). Our objective is to evaluate the association of SAT with risk of treatment failure.

## METHODS

### Study Design and Population

We performed a retrospective cohort study of patients aged ≥18 years with PJI who underwent DAIR. We utilized previously collected data from 3 international cohorts: the United States, Spain, and the Netherlands (NED). The PJI cases from Europe occurred between 2005 and 2016, while the US cases were from 2008 to 2018. We included patients with acute hip or knee PJI who received at least 12 weeks of systemic antibiotic therapy. We excluded patients with chronic infections, especially with sinus tracts; those not treated with DAIR; and those for whom follow-up was <12 weeks from debridement (eg, due to death, loss to follow-up, treatment failure). We also excluded those who refused research authorization. These studies were deemed exempt by institutional review boards.

### Definitions

The definition of PJI was based on criteria from the Musculoskeletal Infection Society [7]. Early and late acute PJIs were included. We defined early acute PJIs as infections occurring within 3 months of the index or revision arthroplasty. Late acute PJIs were defined as infections occurring after 3 months but with a symptom duration <3 weeks prior to debridement. Arthroplasties were primary or revision. SAT was defined as antibiotic therapy after 12 weeks of therapy. In the US cohort, SAT was prescribed as a routine practice in every patient. In the European cohorts, SAT was prescribed at the discretion of the treating physician based on risk of relapse of infection. Some of the factors considered were persistently elevated C-reactive protein and delayed wound healing. The primary outcome was treatment failure, defined as recurrence of PJI, unplanned reoperation secondary to infection, or infection-related death. Covariates such as age, comorbidities, culture results, and surgical history were assessed at the time of debridement.

### Statistical Analysis

The association between SAT and treatment failure was assessed in a multivariable Cox proportional hazards model. The start date for analysis was 12 weeks after surgery. To address potential immortal time bias, which can arise when measuring the effect of an exposure that varies over time in cohort studies, we assessed SAT as a time-varying covariate. We performed least absolute shrinkage and selection operator (LASSO) to reveal important covariates with the most potential for confounding. We selected the penalty parameter for the LASSO model using cross-validation and chose the lambda with the smallest cross-validation c-index. We also considered the largest lambda within 1 SD of the smallest cross-validation c-index, but we felt that this created too much regularization as it selected only a single variable into the model. In addition to our analysis with SAT as a time-varying covariate, we performed a Cox model analysis with country cohorts instead of SAT and with the rest of the covariates unchanged. This analysis allows us to determine if variations in treatment regimens across countries, including differences in how SAT is utilized, lead to different rates of treatment failure. We checked for violations of the proportional hazards assumption using the test proposed by Grambsch and Therneau [8].

## RESULTS

There were 510 patients in the final analysis: 184 from the United States, 236 from NED, and 90 from Spain. The majority were female (58%, n = 296) with a mean age of 70.4 years (SD, 11). Diabetes mellitus was the most common comorbidity with a prevalence of 20% (n = 102). There were 254 knee and 256 hip PJIs. Primary arthroplasties represented 69% of the cases (n = 352). The majority of cases were early acute infections (62%, n = 365) while the mean duration of symptoms was 6.8 days (SD, 7). *Staphylococcus aureus* was the most common causative microorganism (38%, n = 194). For a complete overview of the clinical characteristics of the cohort, see Table 1 and Supplementary Table 1.

**Table 1.** Clinical Characteristics of the Cohort (N = 510)

Variable	No. (%)
Age, y, mean (SD)	70.4 (11)
Sex	
Female	294 (58)
Male	216 (42)
Body mass index, ^a^ mean (SD)	32.1 (7.6)
Joint	
Knee	254 (50)
Hip	256 (50)
Arthroplasty	
Primary	351 (69)
Cemented	392 (77)
Comorbidities	
Diabetes mellitus	101 (20)
Heart failure	55 (11)
Rheumatoid arthritis	48 (9)
Chronic obstructive pulmonary disease	43 (8)
Chronic kidney disease	31 (6)
Liver cirrhosis	9 (2)
Alcohol use ^b^	88 (21)
Active smoking ^c^	57 (14)
History of fracture	50 (10)
Infection type	
Monomicrobial	327 (64)
Polymicrobial	160 (31)
Bacteremia	78 (15)
Microbial etiology	
*Staphylococcus aureus*	193 (38)
Coagulase-negative staphylococci	146 (29)
*Streptococcus* species	97 (19)
*Enterococcus* species	59 (12)
Other gram-positive bacteria	92 (18)
Gram-negative bacteria	88 (17)
Anaerobic bacteria	12 (2)
Therapy	
Rifampin use	282 (55)
Quinolone use	221 (43)
Modular component exchange	239 (47)

^a^Missing in 25 cases.

^b^Denominator was 410.

^c^Denominator was 411.

### SAT and Outcomes

**Figure 1.**
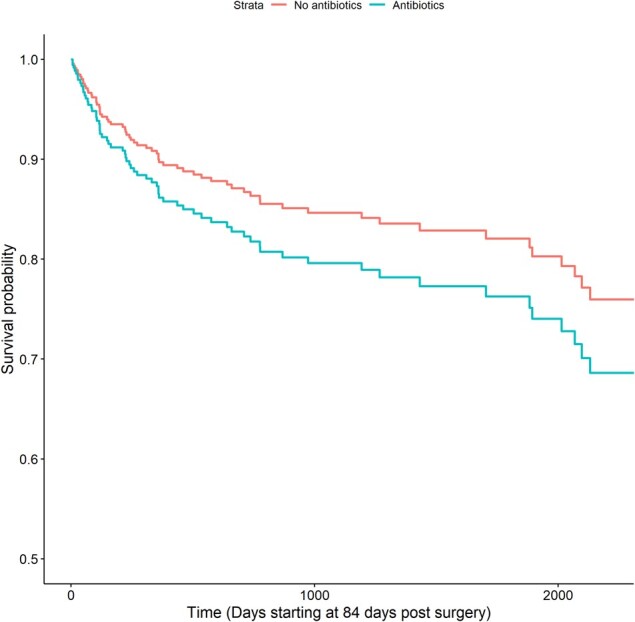
Estimated survival curve for treatment failure on and off Suppressive Antibiotic Therapy based on Cox model.

There were 66 treatment failures (13%), and the median follow-up was 801 days (range, 91–4100). Treatment failure occurred in 39 of 167 patients undergoing SAT, as compared with 27 of 343 patients not receiving SAT. The LASSO model revealed age, infection with *S aureus*, knee joint, late acute infection, chronic kidney disease, revision arthroplasty, and cemented arthroplasty as potential covariates. SAT did not demonstrate a significant impact on the treatment outcome after adjusting for several covariates identified by the LASSO model (hazard ratio [HR], 1.37; 95% CI, .79–2.39; *P* = .27; Figure 1). Subgroup analyses, segmented by joint and infection type (early or late acute), did not demonstrate benefit. The NED cohort showed increased treatment failure with SAT, likely reflecting selection bias (HR, 11.46; 95% CI, 2.08–63.03; *P* < .01). There was no significant relationship between SAT in the US or Spain cohort. Table 2 lists the rest of the HRs.

**Table 2.** Analysis of Treatment Outcomes With Suppressive Antibiotic Therapy as a Time-Varying Covariate

Group	No. of Patients	No. of Events	HR (95% CI)	*P* Value
Overall	510	66	1.37 (.79–2.39)	.27
PJI				
Knee	255	49	1.38 (.72–2.65)	.33
Hip	255	17	0.87 (.25–3.02)	.83
Knee after 180 d	236	37	1.02 (.50–2.10)	.95
Acute infection				
Early	367	30	1.41 (.63–3.17)	.40
Late	143	36	0.95 (.42–2.19)	.91
Cohort				
United States	184	36	0.36 (.11–1.15)	.09
Netherlands	236	16	11.46 (2.08–63.03)	.01
Spain	90	14	0.62 (.10–3.93)	.61

Adjusted for age, *Staphylococcus aureus* infection, type of joint, type of infection, chronic kidney disease, type of procedure, and cemented arthroplasty as appropriate.

Abbreviations: HR, hazard ratio; PJI, periprosthetic joint infection.

### Country Cohort as SAT Proxy

Given the extreme differences in SAT duration among country cohorts, particularly the United States and NED, including SAT and country in the same model would introduce multicollinearity (Supplementary Table 2). As an example of this difference, 167 patients were undergoing SAT for the entire duration of follow-up, and 160 of these were from the US cohort. Additionally, 99 patients were not receiving SAT as of 12 weeks after surgery, and 98 of these were from the NED cohort. Furthermore, the majority of treatment failures were from the US cohort (36/66). Therefore, we conducted an additional sensitivity analysis using the country as a proxy for antibiotic exposure. Controlling for country cohort allowed us to see if differences in country treatment regimens, which include differences in SAT use, resulted in different rates of treatment failure. The NED cohort's HR was 31% lower as compared with the US cohort, though this was not statistically significant (95% CI, .35–1.38; *P* = .30). Similarly, the HR for the Spanish cohort was 11% lower but also nonsignificant (95% CI, .45–1.77; *P* = .74). In addition to reporting the analysis based on a multivariate Cox model, we present the Kaplan-Meier survival curve estimates for each country in Supplementary Figure 1.

## DISCUSSION

There is intense interest in improving the success rates of DAIR for the management of acute PJI. SAT after the initial 12 weeks of antibiotic therapy is proposed as one of the strategies to improve patient outcomes [9]. Unfortunately, the current literature is inconclusive and does not address immortal time bias [10]. Adverse drug reactions and antibiotic resistance are also concerns with SAT [11]. The primary objective of our study was to investigate the utility of SAT following acute PJI managed with DAIR. In our international multicenter retrospective study, there was no significant association between SAT and treatment failure (HR, 1.37; 95% CI, .79–2.39; *P* = .27). The subgroup analysis for joint, type of infection, and country cohort did not show benefit of SAT.

Our findings contribute to the ongoing debate regarding the role of SAT in acute PJI managed with DAIR. This study raises questions about routine SAT, especially considering the risks associated with prolonged antibiotic therapy. We found that the majority of patients remain infection free after 12 weeks without SAT, which is consistent with other studies on DAIR from countries without routine SAT use [12–14]. While we did not see an outright benefit of routine SAT use, the trend is toward benefit for the US cohort (HR, 0.36; 95% CI, .11–1.15). The analysis on outcomes of cohorts revealed that the US cohort had the highest risk of failure when compared with European groups. A review of differences in the characteristics of the country cohorts showed that the US cohort had a longer duration of symptoms and greater body mass index, as well as higher proportions of bacteremia, rheumatoid arthritis, and chronic obstructive pulmonary disease. This suggests that the other uncontrolled and unmeasured covariates might have contributed to poorer outcomes in the US cohort in which SAT might be appropriate.

We note that the Cox model results for the model with SAT as a time-varying covariate and the model with country cohort as a covariate should not be interpreted as implying a causal effect of SAT on treatment failure. This is for 2 reasons. The first is that we believe that there are many differences in treatment regimens among countries that go beyond the variables in our data set and can act as potential confounders of the relationship between SAT and treatment failure. The second involves the well-known issues with trying to interpret parameters from the Cox model as showing causal associations [15]. We do believe that the associations in our results are important because they suggest that patients from the NED cohort have better outcomes than those in the US cohort, even with shorter SAT duration. However, shorter SAT duration may not be the cause of this improvement.

Our subgroup analyses offered a glimpse into possible risk stratification for SAT. Patients without high-risk features—such as *S aureus* infection, knee PJI, late acute infection, chronic kidney disease, revision arthroplasty, and cemented arthroplasty—have the least benefit for SAT. This is consistent with previous literature reporting that these are risk factors for treatment failure in DAIR [16]. Note that most of these studies analyzed the risk factors for patients whose treatment had already failed during antibiotic therapy. Therefore, more studies are necessary to assess the risk factors for failure after 12 weeks of antibiotic therapy. Some experts recommend shared decision making in recommending SAT to patients with factors that are more subjective. These include limited surgical options, recurrent infections, difficult-to-treat pathogens, or immunosuppression [17].

There are some limitations to our study. Ideally, we would exclude those patients whose indication for SAT was a concern for persistent infection, but this was not possible in the US cohort. Therefore, we included patients in the NED and Spanish cohorts whose indication for SAT was a concern for persistent infection, which introduced selection bias against SAT when applied to all cohorts collectively. While this is the largest study to date, the low number of patients limited the power of our analysis, and the few events precluded us from analyzing more covariates. For instance, we were unable to consider variations in initial antibiotic treatment or conduct subgroup analyses for different microbial causes, as the smaller sample sizes would not provide meaningful results. While we made efforts to adjust for potential confounders, differences in comorbidities among cohorts are likely to be residual confounders. Differences in the time frame of the cohorts also introduce variability that might affect the outcomes. Due to limitations in our data, we were unable to perform other statistical methods that can result in parameters with stronger causal implications, such as the G-formula and inverse probability weighting methods.

Given the nonconclusive nature of our findings and the mixed results of prior studies, there is a clear need for further research. Randomized controlled trials would provide more definitive evidence on SAT's role in acute PJI after DAIR management. Additionally, future research should focus on identifying patients who benefit the most from SAT, including the role of artificial intelligence in this regard.

## CONCLUSION

While routine SAT has been viewed as a potential strategy to bolster the success rates of DAIR in managing PJI, our study suggests that its benefits might be limited. There might be a subset of patients who benefit from SAT and should be the focus of future research.

## Supplementary Material

ofae216_Supplementary_Data

## Supplementary Data

Supplementary materials are available at *Open Forum Infectious Diseases* online. Consisting of data provided by the authors to benefit the reader, the posted materials are not copyedited and are the sole responsibility of the authors, so questions or comments should be addressed to the corresponding author.

## Notes

***Acknowledgments.*** The text of this article was reviewed and edited for grammar and clarity with the help of ChatGPT-4.

***Patient consent statement.*** Data collection for the European centers was approved by the ethical committees of the University Medical Center Groningen and Hospital Clinic of Barcelona. This study was deemed exempt by the Mayo Clinic Institutional Review Board.

***Financial support*.** This work was supported by the National Institutes of Health’s National Center for Advancing Translational Sciences (grant UL1TR002494).

## References

[1] Nelson SB, Pinkney JA, Chen AF, Tande AJ. Periprosthetic joint infection: current clinical challenges. Clin Infect Dis 2023:77:e34–45. 37434369 10.1093/cid/ciad360 PMC11004930

[2] Patel R . Periprosthetic joint infection. N Engl J Med 2023; 388:251–62. 36652356 10.1056/NEJMra2203477

[3] Nandi S, Doub JB, DePalma BJ, et al Suppressive antibiotic therapy after debridement, antibiotics, and implant retention (DAIR) is well-tolerated without inducing resistance: a multicenter study. J Arthroplasty 2024:39:795–800. 37717831 10.1016/j.arth.2023.09.004

[4] Malahias MA, Gu A, Harris EC, et al The role of long-term antibiotic suppression in the management of peri-prosthetic joint infections treated with debridement, antibiotics, and implant retention: a systematic review. J Arthroplasty 2020; 35:1154–60. 31955984 10.1016/j.arth.2019.11.026

[5] Tai DBG, Berbari EF, Suh GA, Lahr BD, Abdel MP, Tande AJ. Truth in DAIR: duration of therapy and the use of quinolone/rifampin-based regimens after debridement and implant retention for periprosthetic joint infections. Open Forum Infect Dis 2022; 9:ofac363. 36072695 10.1093/ofid/ofac363 PMC9439576

[6] Bernard L, Arvieux C, Brunschweiler B, et al Antibiotic therapy for 6 or 12 weeks for prosthetic joint infection. N Engl J Med 2021; 384:1991–2001. 34042388 10.1056/NEJMoa2020198

[7] Parvizi J, Zmistowski B, Berbari EF, et al New definition for periprosthetic joint infection: from the workgroup of the musculoskeletal infection society. Clin Orthop 2011; 469:2992–4. 21938532 10.1007/s11999-011-2102-9 PMC3183178

[8] Grambsch PM, Therneau TM. Proportional hazards tests and diagnostics based on weighted residuals. Biometrika 1994; 81:515–26.

[9] Shah N, Hersh B, Kreger A, et al Benefits and adverse events associated with extended antibiotic use in total knee arthroplasty periprosthetic joint infection. Clin Infect Dis 2020; 70:559–65. 30944931 10.1093/cid/ciz261 PMC7768747

[10] Cortés-Penfield NW, Hewlett AL, Kalil AC. Adjunctive rifampin following debridement and implant retention for staphylococcal prosthetic joint infection: is it effective if not combined with a fluoroquinolone? Open Forum Infect Dis 2022; 9:ofac582. 36504699 10.1093/ofid/ofac582 PMC9728514

[11] Vollmer NJ, Rivera CG, Stevens RW, et al Safety and tolerability of fluoroquinolones in patients with staphylococcal periprosthetic joint infections. Clin Infect Dis 2021; 73:850–6. 33606003 10.1093/cid/ciab145

[12] Espíndola R, Vella V, Benito N, et al Rates and predictors of treatment failure in *Staphylococcus aureus* prosthetic joint infections according to different management strategies: a multinational cohort study—the ARTHR-IS Study Group. Infect Dis Ther 2022; 11:2177–203. 36242742 10.1007/s40121-022-00701-0 PMC9669291

[13] Jacobs AME, Valkering LJJ, Bénard M, Meis JF, Goosen JHM. Evaluation one year after DAIR treatment in 91 suspected early prosthetic joint infections in primary knee and hip arthroplasty. J Bone Jt Infect 2019; 4:238–44. 31700773 10.7150/jbji.37757 PMC6831808

[14] Rahardja R, Zhu M, Davis JS, Manning L, Metcalf S, Young SW. Success of debridement, antibiotics, and implant retention in prosthetic joint infection following primary total knee arthroplasty: results from a prospective multicenter study of 189 cases. J Arthroplasty 2023:38(7 suppl 2):S399–404. 37084921 10.1016/j.arth.2023.04.024

[15] Hernán MA . The hazards of hazard ratios. Epidemiology 2010; 21:13–5. 20010207 10.1097/EDE.0b013e3181c1ea43 PMC3653612

[16] Bernaus M, Auñón-Rubio Á, Monfort-Mira M, et al Risk factors of DAIR failure and validation of the KLIC score: a multicenter study of four hundred fifty-five patients. Surg Infect 2022; 23:280–7. 10.1089/sur.2021.320 35172116

[17] Cortes-Penfield N, Krsak M, Damioli L, et al How we approach suppressive antibiotic therapy (SAT) following debridement, antibiotics, and implant retention for prosthetic joint infection. Clin Infect Dis 2024:78:188–98. 37590953 10.1093/cid/ciad484

