# Supplementary material for: Role of Routine Suppressive Antibiotic Therapy After Debridement, Antibiotics, and Implant Retention for Acute Periprosthetic Joint Infections
Source: Open Forum Infect Dis. 2024 Apr 17;11(5):ofae216. doi: 10.1093/ofid/ofae216 (PMC11109604; doi:10.1093/ofid/ofae216)
Supplement: ofae216_Supplementary_Data [file ofae216_supplementary_data.zip › 20240328 DAIR SAT Supp Tables.docx]

Supplemental Table 1: Characteristics of cohorts by country

|  | USA  (N=184) | NED  (N=236) | ESP  (N=90) | p-value |
| --- | --- | --- | --- | --- |
| Mean age (SD) – year | 69 (11) | 71 (11) | 72 (9) | 0.098 |
| Sex – no. (%) |  |  |  | < 0.001 |
| Female | 84 (46) | 152 (64) | 58 (64) |  |
| Male | 100 (54) | 84 (36) | 32 (36) |  |
| Body mass index – mean (SD) ^a^ | 34 (9) | 30 (6) | 32 (6) | < 0.001 |
| Knee joint – no. (%) | 112 (61) | 64 (27) | 78 (87) | < 0.001 |
| Primary arthroplasty – no. (%) | 98 (53) | 191 (81) | 62 (69) | < 0.001 |
| Cemented arthroplasty – no. (%) | 126 (68) | 192 (81) | 74 (82) | 0.003 |
| Mean duration of symptoms – days (SD) | 8.6 (9.4) | 6.3 (5.7) | 4.1 (3) | < 0.001 |
| Comorbidities – no. (%) |  |  |  |  |
| Diabetes | 45 (24) | 43 (18) | 13 (14) | 0.18 |
| Heart Failure | 29 (16) | 16 (7) | 10 (11) | 0.013 |
| Rheumatoid arthritis | 25 (14) | 22 (9) | 1 (1) | 0.004 |
| COPD | 32 (17) | 2 (1) | 9 (10) | < 0.001 |
| Chronic kidney disease | 8 (4) | 20 (8) | 3 (3) | 0.104 |
| Liver cirrhosis | 4 (2) | 0 | 5 (6) | 0.003 |
| Alcohol use ^b^ | 4 (2) | 84 (42) | 0 | < 0.001 |
| Active smoking ^c^ | 19 (10) | 33 (16) | 5 (18) | < 0.001 |
| History of fracture | 16 (9) | 29 (12) | 5 (6) | 0.154 |
| Infection Type – no. (%) |  |  |  |  |
| Monomicrobial | 136 (74) | 131 (56) | 60 (67) | < 0.001 |
| Polymicrobial | 32 (17) | 101 (43) | 27 (30) | < 0.001 |
| Late acute | 110 (60) | 18 (8) | 17 (19) | < 0.001 |
| Bacteremia | 50 (27) | 16 (7) | 12 (7) | < 0.001 |
| Microbial etiology – no. (%) |  |  |  |  |
| *Staphylococcus aureus* | 70 (38) | 92 (39) | 31 (34) | 0.750 |
| Coagulase-negative staphylococci | 37 (20) | 79 (33) | 30 (33) | 0.006 |
| *Streptococcus* species | 41 (22) | 50 (21) | 6 (7) | 0.004 |
| *Enterococcus* species | 9 (5) | 41 (17) | 9 (10) | < 0.001 |
| Other Gram-positive bacteria | 28 (15) | 51 (22) | 13 (14) | 0.149 |
| Gram-negative bacteria | 16 (9) | 39 (17) | 33 (37) | < 0.001 |
| Anaerobic bacteria | 7 (4) | 5 (2) | 0 | 0.141 |
| Therapy – no. (%) |  |  |  |  |
| Rifampin use | 81 (44) | 146 (62) | 55 (61) | 0.001 |
| Quinolone use | 40 (22) | 114 (48) | 67 (74) | < 0.001 |
| Modular component exchange | 111 (60) | 57 (24) | 71 (79) | < 0.001 |
| Mean follow-up time - days (SD) | 1,174 (925) | 840 (687) | 1,493 (958) | ± |
| Mean time on SAT - days (SD) | 1,058 (880) | 20 (66) | 138 (266) | ± |

Abbreviations: COPD, chronic obstructive pulmonary disease; ESP, Spain; NED, Netherlands; SAT, suppressive antibiotic therapy; SD, standard deviation; USA, United States of America

^a^ Denominators for USA, NED and ESP cohorts were 183, 225 and 77, respectively.

^b^ Denominators for NED and ESP cohorts were 200 and 27, respectively.

^c^ Denominators for NED and ESP cohorts were 200 and 26, respectively.

^d^ Denominators for USA, NED and ESP cohorts were 197, 309, and 95, respectively. These figures represent the total number of antibiotic courses administered. The count may exceed the actual size of each cohort since some individuals received multiple courses of antibiotics.

^±^P-value inappropriate due to censoring and different rate of events

Supplemental Table 2: Proportion of events on and off suppressive antibiotic therapy

|  | USA  (N=184) | | NED  (N=236) | | ESP  (N=90) | |
| --- | --- | --- | --- | --- | --- | --- |
|  | No failure | Failure | No failure | Failure | No failure | Failure |
| On SAT | 128 | 32 | 0 | 5 | 0 | 2 |
| Off SAT | 20 | 4 | 220 | 11 | 76 | 12 |

Abbreviations: ESP, Spain; NED, Netherlands; SAT, suppressive antibiotic therapy; USA, United States of America
