# Supplementary figures and images for: Role of Routine Suppressive Antibiotic Therapy After Debridement, Antibiotics, and Implant Retention for Acute Periprosthetic Joint Infections
Source: Open Forum Infect Dis. 2024 Apr 17;11(5):ofae216. doi: 10.1093/ofid/ofae216 (PMC11109604; doi:10.1093/ofid/ofae216)

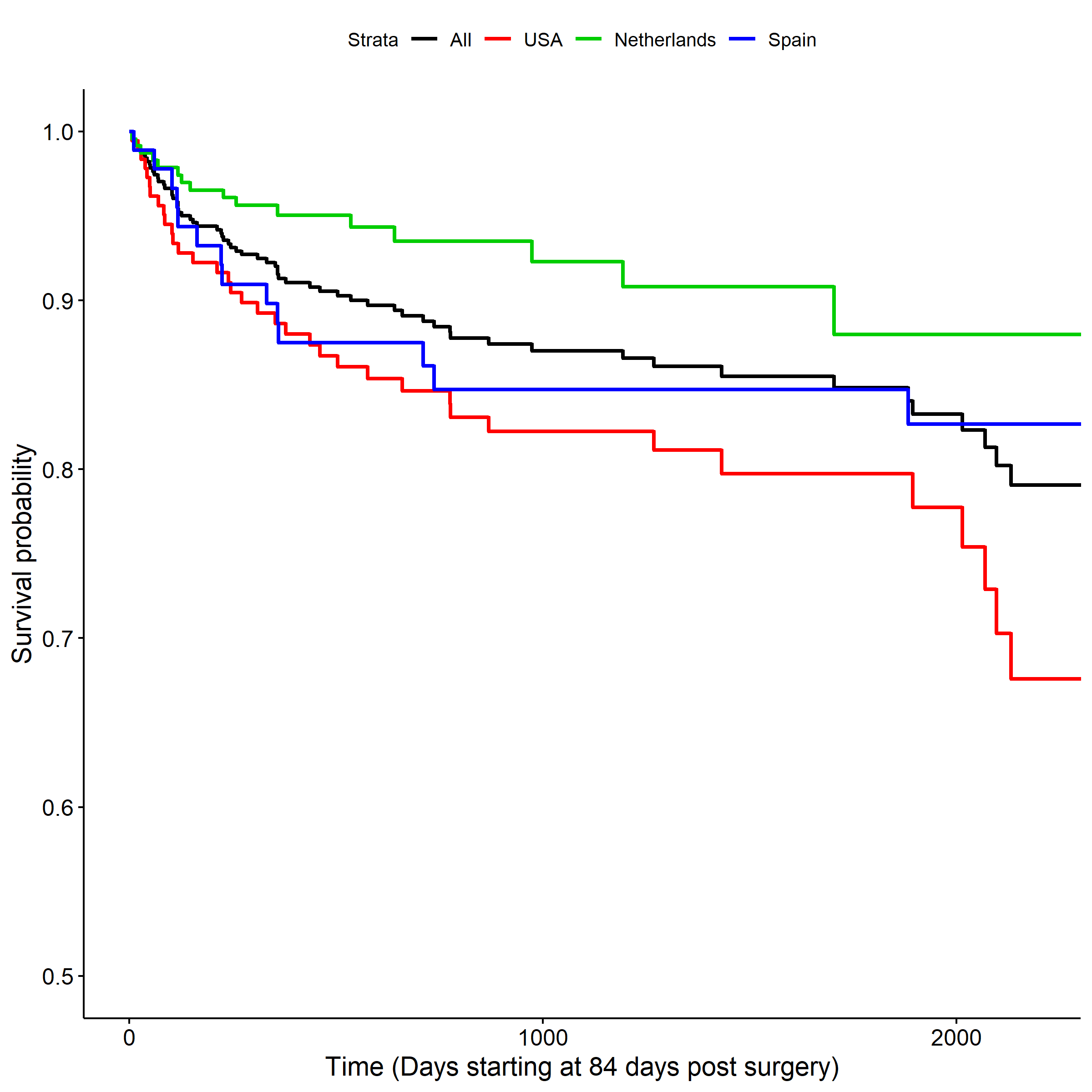

Supplement: ofae216_Supplementary_Data [file ofae216_supplementary_data.zip › 20240404 DAIR SAT Supp Fig 1.tif]
